# A Case of Anti-SAE1 Dermatomyositis

**DOI:** 10.1155/2022/9000608

**Published:** 2022-03-04

**Authors:** Max de Vries, Marco W. J. Schreurs, Els J. M. Ahsmann, Marcela Spee-Dropkova, Faiz Karim

**Affiliations:** ^1^Department of Internal Medicine, Groene Hart Hospital, Bleulandweg 10 2803 HH, Gouda, Netherlands; ^2^Department of Immunology, Erasmus MC, University Medical Center, Postbus 2040 3000 CA, Rotterdam, Netherlands; ^3^Department of Clinical Pathology, Groene Hart Hospital, Bleulandweg 10 2803 HH, Gouda, Netherlands; ^4^Department of Radiology, Groene Hart Hospital, Bleulandweg 10 2803 HH, Gouda, Netherlands

## Abstract

**Introduction:**

Anti-SAE1 antibodies have a low prevalence in dermatomyositis patients. *Case Description*. A 61-year-old woman presented with progressive shortness of breath, arthralgia, heliotrope rash, Gottron's papules, and erythematous rash. She had an interstitial lung disease (ILD) with a significant decrease in lung function. There was no muscle involvement. Immunological laboratory test results showed strongly positive anti-SAE1 antibodies. Glucocorticoid treatment resulted in remission of dermatomyositis.

**Conclusion:**

Anti-SAE antibodies in dermatomyositis patients are closely linked to unique clinical features.

## 1. Introduction

Dermatomyositis is an idiopathic inflammatory myopathy with a wide variability in clinical presentation due to a range of cutaneous and systemic manifestations. This includes variety in muscle and joint manifestation, pulmonary manifestation, and association with malignancy. This inconsistency creates a diagnostic challenge for physicians. Myositis-specific autoantibodies (MSAs) are antibodies which are only found in inflammatory myopathies, and some MSAs are specific for dermatomyositis [1]. Every MSA is closely linked to both unique clinical features, response to treatment and prognosis [2]. In 2007, antismall ubiquitin-like modifier 1-activating enzyme (SAE1) antibodies were identified as a dermatomyositis-specific MSA [3]. Its frequency among dermatomyositis patients is very low, ranging from 1.5% to 8.0% [4]. As few patients with anti-SAE1 dermatomyositis have been described, it is important to describe more cases of this rare subtype. We describe one patient with anti-SAE1 dermatomyositis.

## 2. Case Presentation

**Figure 1:**
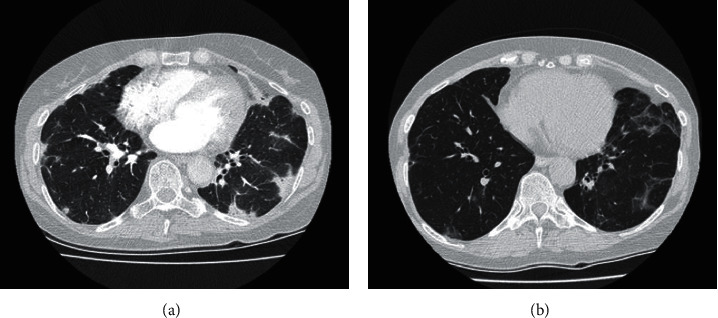
Imaging studies of the patient. (a) CT-thorax 2018 (pretreatment): bilateral patchy consolidations with distortion and dilatation of the bronchioles and parenchymal distortion with peripheral subpleural and peribronchovascular distribution, which are compatible with an organizing pneumonia. (b) CT-thorax 2019 (posttreatment): bilateral atelectasis with traction bronchiectasis, which are compatible with residual abnormalities after an organizing pneumonia.

The patient characteristics and the main clinical features are given in Table 1. A 61-year-old Caucasian woman with a medical history of hypertension, interstitial cystitis, and anteroseptal myocardial infarction presented to our outpatient clinic. For her comorbidities, she used the following medication: carbasalate calcium, perindopril, nitrofurantoin, celecoxib, and amitriptyline. She presented with progressive shortness of breath, fatigue, arthralgia, and skin alterations. The skin alterations were the initial complaints; she suffered from and was thought to have antibiotic hypersensitivity. She was treated with amoxicillin clavulanate for pneumonia. However, skin alterations persisted after discontinuation of the antibiotics. She had no complaints of dysphagia, muscle pain, or proximal muscle weakness. Her vital signs were normal. Physical examination revealed a heliotrope rash and Gottron's papules on the dorsal metacarpophalangeal and proximal interphalangeal joints and an erythematous rash on her back and upper leg. Laboratory test results are given in Table 1. Pulmonary function testing showed a vital capacity of 2.08 L, which was 70% of the predicted value, and a diffusing capacity for carbon monoxide of 54% of the predicted value. CT scan showed diffuse consolidations, suspect for interstitial lung disease (ILD) (Figure 1(a)).

**Figure 2:**
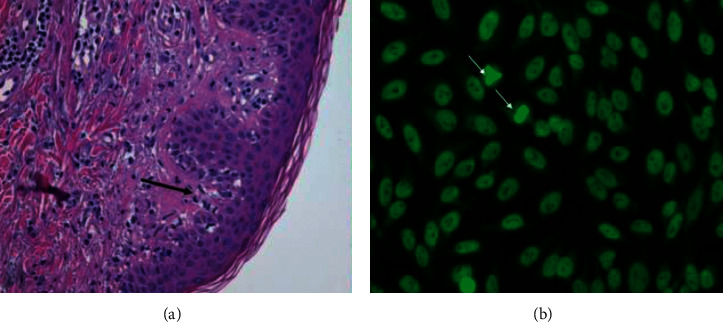
Skin biopsy and ANA determination. (a) H&E staining showing vacuolar changes in the basal layer of the epidermis (arrow) and perivascular lymphocytic infiltration of the dermis. (b) The IIF HEp-2 pattern showing a positive ANA with combined nuclear dense (AC-2) and fine speckled (AC-4) pattern (http://www.ANApatterns.org), the latter being compatible with anti-SAE1 reactivity. Arrows indicate fine speckled nucleoplasm in mitotic cells outside chromatin mass.

Skin biopsy hematoxylin and eosin (H&E) staining showed vacuolar changes in the basal layer of the epidermis and mild perivascular lymphocytic infiltration of the dermis (Figure 2(a)). Direct immunofluorescence showed depositions of IgA, IgG, and IgM in the basal membrane zone which is compatible with dermatomyositis and cutaneous lupus erythematosus. Immunological laboratory test results showed strongly positive anti-SAE1 antibodies. The antinuclear antibodies (ANA) determination by indirect immunofluorescence (IIF) on HEp-2 cells is shown in Figure 2(b). This shows a positive ANA with both nuclear dense speckled (AC-2) and nuclear fine speckled (AC-4) pattern, the latter being compatible with anti-SAE1 reactivity.

Our patient was admitted to the hospital for methylprednisolone treatment (1000 mg a day for three days). An abdominal ultrasound was preformed to screen for malignancy and because of the elevated liver enzymes, but no abnormalities were found. After three days of treatment with methylprednisolone, we started prednisolone 60 mg every day, which eventually was tapered down to 5 mg every day. She was shortly treated with azathioprine. This was discontinued due to thrombocytopenia. Monotreatment with low-dose prednisone 5 mg/day was, however, sufficient. The elevated liver enzymes abated during treatment. The dermatomyositis is in remission. Last control was in November 2021 (2 years after start of treatment).

## 3. Discussion

Anti-SAE1 antibodies are relatively rare in dermatomyositis patients with a very low prevalence ranging between 1.5 and 8.0%, and only few patients have been described so far. There is heterogeneity in clinical features among patients. Moreover, there seems to be difference in clinical presentation between Western and Asian populations, which creates a diagnostic challenge for clinicians [5].

Cutaneous disease was the initial manifestation of the dermatomyositis in our patient, which is a frequent finding in anti-SAE1-positive dermatomyositis patients. However, there is also a subset which presents with skin and muscle disease simultaneously. Typical findings in anti-SAE1 positive patients are heliotrope rash, Gottron's sign, and papules. An erythematous rash is also described in a subset of patients [1]. Dysphagia is present in 47% of patients [5].

The prevalence of ILD among anti-SAE1 positive patients is nearly 50% and is usually mild, with few patients having respiratory complaints despite having abnormalities on imaging, which are compatible with ILD. However, our patient did experience respiratory symptoms and had significant decrease in lung function. The characteristics of ILD in anti-SAE1 patients on chest HRCT are subpleural peripheral-dominant small ground glass opacities, corresponding to organizing pneumonia [2].

As other MSAs, anti-SAE1 antibodies may be associated with an increased risk of cancer. Therefore, tumor screening is recommended after initial diagnosis in all dermatomyositis patients [6]. Most studies on anti-SAE1 antibodies and cancer show a positive association [5, 7–9]. All anti-SAE1 positive patients who developed cancer suffered from adenocarcinoma. These adenocarcinomas were from cervical, pulmonary, esophageal, or rectal origin. The frequency of cancer in anti-SAE1-positive patients in studies included by Lu et al. ranged between 14% and 57% [10]. However, a common denominator of these studies was a small sample size.

Glucocorticoid treatment is the preferred treatment in dermatomyositis patients. Both monotherapy with glucocorticoids as combined treatment with glucocorticoid and immunosuppressive agents (methotrexate, cyclophosphamide, cyclosporine, tacrolimus, rituximab, thalidomide, or immunoglobulins) are described as treatment for anti-SAE1 positive patients [4].

In conclusion, anti-SAE1 antibodies are relatively rare in dermatomyositis patients. Classical findings in anti-SAE1 positive patients are heliotrope rash, Gottron's sign, and papules, muscle weakness, and dysphagia. Additional findings include mild interstitial lung disease, and there may be an increased risk of cancer. Glucocorticoid treatment is the preferred first-line treatment in dermatomyositis patients.

## Figures and Tables

**Table 1:** Characteristics and the main clinical features of the patient.

Gender	Female
Age	61 years

Medical history	Hypertension, interstitial cystitis, and anteroseptal myocardial infarction

Symptoms	Progressive shortness of breath, fatigue, arthralgia, and skin alterations

Physical examination	Heliotrope rash, Gottron's papules on the dorsal metacarpophalangeal and proximal interphalangeal joint, and an erythematous rash on back and upper leg. There was no muscle weakness

Laboratory test results	2018: hemoglobin 8.8 mmol/L; leucocytes 4.4 10 *∗* 9/L; CRP 5 mg/L; ASAT 332 IU/L; ALAT 282 IU/L; LDH 377 IU/L; ALP 170 IU/L; GGT 101 IU/L; bilirubin 9 *µ*mol/L; CK 198 IU/L; complement analysis: normal; ANA: positive (nuclear dense speckled (AC-2) and nuclear fine speckled (AC-4) pattern); ANCA (MPO/PR3): normal; rheumatoid factor IgM: normal; CCP antibody: normal

Immunological laboratory tests	Systemic sclerosis antibodies^*∗*^: negative Positive myositis antibodies^*∗*^: anti-SAE1 Negative myositis antibodies^*∗*^: anti-OJ; anti-EJ; anti-PL-12; anti-PL-7; anti-SRP; anti-Jo-1; anti-PM-Scl75; anti-PM-Scl100; anti-Ku; anti-NXP2; anti-MDA5; anti-TIF-1 gamma; anti-Mi-2 beta; anti-Mi-2 alpha

Histology	Skin biopsy: H&E staining showed vacuolar changes in the basal layer of the epidermis and perivascular lymphocytic infiltration of the dermis. Immunofluorescence: dispositions of IgA, IgG, and IgM in the basal membrane which is compatible with dermatomyositis and cutaneous lupus erythematosus

Pulmonary function testing	2018: vital capacity: 2.08 L (70% of the predicted value); diffusing capacity for carbon monoxide: 54% of the predicted value

Imaging	See Figure 1

Treatment	Methylprednisolone (1000 mg a day for three days) followed by prednisolone 60 mg every day, which eventually was tapered down to 5 mg every day. Azathioprine was discontinued because of thrombocytopenia

CRP, C-reactive protein; ASAT, aspartate aminotransferase; ALAT, alanine aminotransferase; LDH, lactate dehydrogenase; ALP, alkaline phosphatase; GGT, gamma-glutamyl transpeptidase; CK, creatinine kinase; ANA, antinuclear antibodies; ANCA, antineutrophil cytoplasmic antibodies; CCP, cyclic citrullinated peptide. ^*∗*^Systemic sclerosis and myositis antibodies were determined by line immune assay (Euroline profile, Euroimmun, Lübeck, Germany, according to manufacturer's instructions).

## Data Availability

The clinical data used to support the findings of this study are included within the article.

## References

[1] Wolstencroft P. W., Fiorentino D. F. (2018). Dermatomyositis clinical and pathological phenotypes associated with myositis-specific autoantibodies. *Current Rheumatology Reports*.

[2] Gono T., Tanino Y., Nishikawa A. (2019). Two cases with autoantibodies to small ubiquitin-like modifier activating enzyme: a potential unique subset of dermatomyositis-associated interstitial lung disease. *International Journal of Rheumatic Diseases*.

[3] Betteridge Z. E., Gunawardena H., Chinoy H. (2009). Clinical and human leucocyte antigen class II haplotype associations of autoantibodies to small ubiquitin-like modifier enzyme, a dermatomyositis-specific autoantigen target, in UK Caucasian adult-onset myositis. *Annals of the Rheumatic Diseases*.

[4] Jia E., Wei J., Geng H. (2019). Diffuse pruritic erythema as a clinical manifestation in anti-SAE antibody-associated dermatomyositis: a case report and literature review. *Clinical Rheumatology*.

[5] Ge Y., Lu X., Shu X., Peng Q., Wang G. (2017). Clinical characteristics of anti-SAE antibodies in Chinese patients with dermatomyositis in comparison with different patient cohorts. *Scientific Reports*.

[6] Schlecht N., Sunderkötter C., Niehaus S., Nashan D. (2020). Update on dermatomyositis in adults. *JDDG: Journal der Deutschen Dermatologischen Gesellschaft*.

[7] Tarricone E., Ghirardello A., Rampudda M., Bassi N., Punzi L., Doria A. (2012). Anti-SAE antibodies in autoimmune myositis: identification by unlabelled protein immunoprecipitation in an Italian patient cohort. *Journal of Immunological Methods*.

[8] Fujimoto M., Matsushita T., Hamaguchi Y. (2013). Autoantibodies to small ubiquitin-like modifier activating enzymes in Japanese patients with dermatomyositis: comparison with a UK Caucasian cohort. *Annals of the Rheumatic Diseases*.

[9] Muro Y., Sugiura K., Akiyama M. (2013). Low prevalence of anti-small ubiquitin-like modifier activating enzyme antibodies in dermatomyositis patients. *Autoimmunity*.

[10] Lu X., Peng Q., Wang G. (2019). The role of cancer-associated autoantibodies as biomarkers in paraneoplastic myositis syndrome. *Current Opinion in Rheumatology*.

